# Fabrication and Appraisal of Simvastatin via Tailored Niosomal Nanovesicles for Transdermal Delivery Enhancement: In Vitro and In Vivo Assessment

**DOI:** 10.3390/pharmaceutics13020138

**Published:** 2021-01-21

**Authors:** Heba F. Salem, Rasha M. Kharshoum, Heba A. Abou-Taleb, Hanan Osman Farouk, Randa Mohammed Zaki

**Affiliations:** 1 Department of Pharmaceutics and Industrial Pharmacy, Faculty of Pharmacy, Beni-Suef University, Shehata Hegazi Street, P.O. Box 62514 Beni-Suef, Egypt; heba_salem2004@yahoo.co.uk (H.F.S.); rasha_mo@yahoo.com (R.M.K.); 2 Department of Pharmaceutics and Industrial Pharmacy, Faculty of Pharmacy, Nahda University (NUB), P.O. Box 62511 Beni-Suef, Egypt; habhob61076@yahoo.com (H.A.A.-T.); hanan_elsahly90@yahoo.com (H.O.F.); 3 Department of Pharmaceutics, Faculty of Pharmacy, Prince Sattam Bin Abdulaziz University, P.O. Box 173, Al-Kharj 11942, Saudi Arabia

**Keywords:** thin-film hydration method, niosomes, factorial design, skin permeation, transdermal delivery

## Abstract

Simvastatin (SIM) is a HMG-CoA reductase inhibitor employed in the management of hyperlipidemia. However, its low bioavailability limits its clinical efficacy. The objective of this study was to overcome the poor bioavailability of SIM via the transdermal application of a SIM-loaded niosomal gel. Niosomes loaded with SIM were fabricated by means of the thin-film hydration method and optimized through a 3^3^-factorial design utilizing Design Expert^®^ software. The prepared niosomes were evaluated for entrapment efficiency (EE%), zeta potential, vesicle size, and cumulative percentage of drug release. The optimum niosomal formulation was loaded on the gel and evaluated for physical properties such as color, clarity, and homogeneity. It was also evaluated for spreadability, and the cumulative % drug release. The best niosomal gel formula was appraised for ex vivo permeation as well as pharmacokinetic study. The SIM-loaded niosomes showed EE% between 66.7–91.4%, vesicle size between 191.1–521.6 nm, and zeta potential ranged between −0.81–+35.6 mv. The cumulative percentage of drug released was ranged from 55% to 94% over 12 h. SIM-loaded niosomal gels were clear, homogenous, spreadable, and the pH values were within the range of physiological skin pH. Furthermore, about 73.5% of SIM was released within 24 h, whereas 409.5 µg/cm^2^ of SIM passed through the skin over 24 h in the ex vivo permeation study. The pharmacokinetic study revealed higher AUC_0–∞_ and Cmax with topical application of SIM-loaded niosomal gel compared to topical SIM gel or oral SIM suspension. The topical application of SIM-loaded niosomal gel ascertained the potential percutaneous delivery of SIM.

## 1. Introduction

Hyperlipidemia refers to high cholesterol levels in the blood. Cholesterol is an essential biological fatty molecule that is produced by the liver and found in all body cells [1]. It is necessary in order for cell membranes to be healthy, as well as for the functions of the brain, and the production of steroid hormones and vitamin D [1,2]. There are two types of lipoproteins that are responsible for conveying cholesterol to the cells—high-density lipoproteins (HDL), or good cholesterol, and low-density lipoproteins (LDL), or bad cholesterol [3]. LDL causes serious effects on health as it permits the extra cholesterol to accumulate in the blood vessel walls, causing atherosclerosis, whereas HDL is very useful for our health because it transfers the surplus cholesterol to the liver to be excreted through bile [4]. Therefore, the synthesis and use of cholesterol must be strongly controlled in order to inhibit abnormal over-deposition within the body (hyperlipidemia), as such deposition ultimately leads to atherosclerosis, which is the main cause of coronary arteries diseases and death [5].

Simvastatin, a HMG-CoA (3-hydroxy-3-methyl-glutaryl-coenzyme A) reductase inhibitor, is used in the treatment of hyperlipidemia [6]. The HMG-CoA reductase is the main enzyme required for cholesterol synthesis [7]. HMG-CoA reductase inhibitors act through competitive inhibition for this enzyme. Therefore, there is a decrease in intracellular cholesterol biosynthesis, which causes an enhancement in the expression of high-affinity receptors for LDL on hepatocyte membranes and simultaneously a drop in the production of LDL-cholesterol [8]. HMG-CoA reductase inhibitors are now mostly prescribed in many countries as antihyperlipidemics due to their lipid-lowering effect [9]. They are the most active agents established today for the treatment of patients suffering from primary and secondary hypercholesterolemia related to high levels of LDL-cholesterol [10]. SIM can reduce LDL-cholesterol by 50% and elevates HDL-cholesterol by 5–10% [10]. SIM has a direct antiatherosclerotic effect on the walls of arteries, beyond its lipid-lowering effects, which leads to a more considerable inhibition of heart disease [11]. Unfortunately, SIM has many limitations. It is subjected to low and incomplete absorption from the gastrointestinal tract due to its poor solubility and is also exposed to hepatic tissue binding and first pass metabolism, resulting in low bioavailability [12]. In addition, it possesses a short biological half-life (1.5–2 h) [13]. All these factors mean that SIM shows low efficacy. However, the transdermal delivery of SIM is a potential application route.

Transdermal delivery has many advantages over the oral route, involving eluding the first pass effect and attaining sustained release by avoiding the problems related to drug absorption from the gastrointestinal tract, such as pH and enzymatic activity [14]. Furthermore, transdermal delivery would reduce the dose frequency and achieve slight variations in the drug plasma level [15]. Furthermore, transdermal delivery is a non-invasive administration route so the drug’s effect can be stopped rapidly by its removal from the surface of the skin [14,16]. However, the stratum corneum limits the absorption and bioavailability of transdermally administered drugs [17]. Several approaches have been established to deliver the drugs successfully through this barrier, involving the use of nanovesicular carriers [18,19].

Niosomes (non-ionic surfactant vesicles), as an innovative nanovesicular drug delivery system, can enhance the stability and solubility of drugs [19]. They have been established to protect drugs from degradation and to deliver drugs to their target organs. Moreover, they extend the circulation time of drugs in the blood [20]. Niosomes are developed upon hydration of lipid and non-ionic surfactants, resulting in a closed bilayer structure [21]. The bilayers have both outer and inner hydrophilic surfaces with a sandwiched lipophilic area in between. Niosomes, as an active drug carrier, have the ability to encapsulate both hydrophobic and lipophobic drugs in the niosomal membrane and niosomal aqueous core, respectively [22]. Niosomes have been suggested for several routes of application such as peroral, intravenous, intramuscular, and transdermal [23].

The main two components used for niosome preparation are lipid materials (cholesterol or l-α-soya phosphatidyl choline) and non-ionic surfactants. The lipid materials are employed to make the niosomal bilayer rigid and provide better stability for the niosomes [24]. Non-ionic surfactants are the most important component for the progress and arrangement of niosomes [21]. Non-ionic surfactants include spans (spans 85, 80, 60, 40, and 20); tweens (tweens 80, 60, 40, and 20); and Brij (35, 58) [19]. the effectiveness of the drug may be improved by these surfactants, possibly by facilitating the drug’s uptake by the target cells (Shilakari et al.) [25].

Niosomes are more advantageous than liposomes due to their higher chemical and physical stability. They evade some disadvantages of liposomes, such as purity variation and high preparation cost of phospholipids [26]. Niosomes are compatible with the human body, biodegradable, and do not cause any immunological reactions [27]. The characteristics of niosome vesicles can be altered by changing the composition and concentration of the niosomes’ components and size, and through the inclusion of surface charge inducing agents [28]. Studies have reported that niosomes augment the drug contact time in the stratum corneum and increase the permeation of the entrapped drugs across the skin. Moreover, they have been found to reduce the adverse reactions and give a substantial drug release [29].

The present study aimed to develop SIM-loaded niosomal nanovesicles embedded in a gel for transdermal delivery in order to enhance the systemic absorption of SIM by avoiding gastrointestinal side effects and the first pass effect. The SIM niosomal formulations were evaluated for different physicochemical properties such as entrapment efficiency, shape and vesicle size, zeta potential, cumulative % of drug release, and ex vivo permeation. Additionally, the pharmacokinetic parameters of the SIM were assessed following oral and topical application.

## 2. Materials and Methods

### 2.1. Materials

Simvastatin (SIM) was kindly donated by Hikma Pharma S.A.E (Cairo, Egypt). Cholesterol and cetyl pyridinium chloride (CPC) were purchased from VWR International Co (Bridgeport, NJ, USA). Span60, Tween 80, and Cremphor RH 40 were obtained from Ruger Chemical Co, Inc. (Linden, NJ, USA). Carbapol 940, hydroxypropyl methyl cellulose (HPMC H_15_), sodium carboxymethylcellulose (Na CMC), polyethylene glycol (PEG 400), propylene glycol, and dialysis bags with a molecular weight cut-off of 12,000 Da were purchased from Sigma-Aldrich (St. Louis, MO, USA). HPLC-grade acetonitrile, methanol and chloroform were purchased from Thermo Fisher Scientific Inc. (Pittsburgh, PA, USA).

### 2.2. Experimental Design

A 3^3^ full factorial design was created using Design Expert software^®^ (version 11.0.6.0, StatEase Inc. Minneapolis, MN, USA) to investigate the effect of three independent factors—the concentration of surfactant (X1), the type of surfactant (X2), and CPC concentration (X3) on the physicochemical properties of SIM niosome formulations. The dependent variables were entrapment efficiency (EE%), vesicle size, zeta potential, and cumulative percentage of SIM released from niosomes after 12 h (Q12h) (Table 1).

### 2.3. Preparation of SIM-Loaded Niosomes

Various SIM niosomal formulations were formulated by means of the thin-film hydration method reported by Bangham et al. [30]. Three non-ionic surfactants, namely, Span 60, Tween 80, and Cremophor RH40, were used in different concentrations along with cholesterol (lipid component). CPC was utilized to impart positive charges for the preparation of positively charged niosomes. The exact weight of the SIM (20 mg) was dissolved in 5 mL methanol. A precise weight of non-ionic surfactant, cholesterol with or without CPC, was fully solubilized in 5 mL chloroform. Both methanol and chloroform solutions were mixed in the rotavapor flask (Hikma Pharma S.A.E, Cairo, Egypt) and allowed to evaporate under reduced pressure, keeping the water bath at 60 °C ± 2 °C. Then, the formed thin film was hydrated using 10 mL phosphate buffer with rotation at 60 °C ± 2 °C. Traces of the non-hydrated film was ultra-sonicated to assure the full hydration of the niosomal film.

### 2.4. Characterization of the Prepared SIM Niosomes

#### Entrapment Efficiency %

The unentrapped SIM was isolated from SIM-loaded niosomes by means of a cooling centrifuge (SIGMA 3–30 K, Sigma, Steinheim, Germany) adjusted to 15,000 rpm for 1 h at 4 °C. The supernatant containing unentrapped SIM was isolated, suitably diluted, and assessed for SIM content by HPLC at λ238 (Thermo Fisher Scientific, Waltham, MA, USA). Each measurement was repeated in triplicate; the following equation was employed to calculate EE% [31]:

Entrapment efficiency (%) = ((C_t_ − C_f_)/ C_t_) × 100 (1)

C_t_ is the total amount of SIM and C_f_ is the amount of free SIM.

### 2.5. Vesicle Size and Size Distribution

The vesicle size for all SIM niosomes formulations N1–N27 (1 mL diluted with 50 mL deionized water) was estimated by dynamic light scattering (DLS) using a particle sizing system (Zeta Potential/Particle Sizer, NICOMP™ 380 ZLS, Entegris Particle Sizing, Santa Barbara, CA, USA). Briefly, the samples were exposed to a laser beam (wavelength λ = 532 nm), the nanovesicles caused scattering for the light and the fluctuation of the scattered light were detected by a photon detector at a scattering angle of 90°. Then, a DSL Digital correlator measured the intensity of the fluctuations by calculating the intensity correlation function, which was then related to the radius of the nanovesicles. The polydispersity index (PDI) was employed as an indicator for the degree of distribution and vesicle size homogeneity. The PDI was estimated to be in accordance with Vora et al. [32]. PDI = standard deviation/mean vesicle diameter. PDI is basically a representation of the distribution of size populations within a given sample. The numerical value of PDI ranges from 0.0 (for a perfectly uniform sample with respect to the particle size) to 1.0 (for a highly polydisperse sample with multiple particle size populations) [33]. All measurements were done in triplicate at 25 °C ± 1 °C.

### 2.6. Zeta Potential

Zeta potential is a predictable measure for the extent of repulsion and attraction between vesicles. So, the benefit of Zeta potential measurements is to improve the niosomes’ stability. The zeta potential of all SIM niosomes formulations (1 mL diluted with 50 mL deionized water) was estimated by a Malvern Zetasizer (Malvern Instruments, Worcestershire, UK). All measurements were done in triplicate at 25 °C ± 1 °C.

### 2.7. In Vitro Release Study

The drug release from different SIM niosome formulations in comparison to pure SIM solution was examined, utilizing a diffusion technique based on cellophane dialysis bags (molecular weight cut off 12,000; Sigma-Aldrich, Cairo, Egypt). The bags were soaked in phosphate-buffered saline (pH 6.8) for 12 h before being mounted. Briefly, SIM niosome formulations and SIM solution (each of them equivalent to 5 mg SIM) were put in the dialysis bags, which were subsequently bound around cylinders. The bags were then placed into beakers containing 50 mL phosphate-buffered saline (pH 6.8) [34] at 37 °C ± 0.5 °C, with continuous and constant stirring at 50 rpm by means of a magnetic stirrer. At prespecified time intervals over 12 h, samples of 2 mL were drawn and restored with an equal volume of fresh dissolution medium to retain the sink condition. All samples were filtered and measured for SIM content by HPLC at λ238. The release study was done in triplicate, and the cumulative percentage of SIM released was represented as an average (±SD).

### 2.8. Optimization of SIM Niosome Formulations

#### 2.8.1. Experimental Model Evaluation

Design Expert^®^ (DE) software was configured for the optimization of the process to operate the desirability index (DI), which displays a range between 0 and 1 representing a satisfactory level for a set of independent variables. A D value of 0 expresses an unacceptable formula, whereas a D value of 1 expresses a highly acceptable formula.

These parameters show the estimated calculus for effects, degrees of freedom, F-ratio, and *p*-value to create a proper design for the applied factors and indications. *p*-values lesser than 0.05 produce notable calculus. Coefficient of determination (R-squared/R2), adjusted R-squared (R2), and predicted R-squared (R2) values were applied to predict the proper design. In this study, we used a specific criterion, pointing out an optimum formulation expressing the minimum vesicle size and the maximum EE%, zeta potential, and % released.

#### 2.8.2. Characterization of the Optimized SIM Niosomal Formulation

The optimized formulation was formulated and assessed for different characteristics such as EE%, vesicle size, zeta potential, and its cumulative percentage of drug release. This was also examined by means of transmission electron microscopy (JEM-1230, Jeol, Tokyo, Japan) in order to observe the surface morphology, as well as being subjected to a stability study. The best formula was selected and ultimately amalgamated into a suitable gel base.

#### 2.8.3. Transmission Electron Microscopy (TEM) for the Optimized Formulation

The morphological appearance of the optimized SIM niosomal formulation was characterized by means of TEM (JEM-1230, Jeol, Tokyo, Japan). The operating accelerating voltage was 80 kV [35]. Firstly, the SIM niosomal formulation was diluted, followed by sonication in an ultrasonic bath (Model 3510; Branson Ultrasonics, Danbury, CT, USA) to decrease the agglomeration of nanovesicles. Later, a drop of this formulation was taken and put on a grid coated with carbon then stained with 2% phosphotungstic acid.

#### 2.8.4. Physical Stability Study for the Optimized Formulation

The optimized niosomal formulation was kept for 3 months at 4 °C. After 30, 60, and 90 days of storage, samples were drawn from the formulation and validated for the vesicles size and EE% [36].

#### 2.8.5. Formulation of SIM-Loaded Niosomal Gel

The optimized SIM niosomal formulation was integrated into different gel bases. Three different polymers—carbapol 940, HPMC H15, and Na CMC—were used in various concentrations for gel preparations.

The exact weight of the polymer was sprinkled in a small quantity of distilled water, mixed well, and kept for 4–5 h. Then, the optimized SIM niosomal formulation (equivalent to 20 mg SIM) was centrifuged, and the obtained pellets were dispersed into the polymer dispersion with constant stirring by a magnetic stirrer (Nickel Electro™ CH-1E; Nickel-Electro Ltd., Weston-super-Mare, UK) to inhibit the development of any aggregations and to result in a smooth uniform dispersion [37]. The pH of the carpobol dispersion was adjusted by the addition of 1% (*w*/*v*) triethanolamine solution as a neutralizing agent [37]. Finally, the weight of the gel was adjusted to 10 g by the addition of distilled water.

### 2.9. Characterization of SIM-Loaded Niosomal Gel Formulations

#### 2.9.1. Physical Parameters

The prepared gels were inspected visually for different physical parameters such as clarity, homogeneity, phase separation, color, and odor [38].

#### 2.9.2. pH Measurements

The pH of different SIM-loaded niosomal gel formulations was assessed by means of a digital pH meter (3505pHmeter, Jenway, Staffordshire, UK), using the procedure formerly reported by Aly et al. [39]. One gram of the gel was diluted and dispersed well in 10 mL distilled water, then pH was measured in triplicate, followed by calculating the average reading for each formula.

#### 2.9.3. Spreadability

The spreadability of the SIM-loaded niosomal gel was assessed by putting 0.5 g of the gel in the center of a watch glass (5 cm in diameter), and then another watch glass was gently placed over the first one and left for 5 min to make sure that there was no expectation of further spreading. Diameters of the spread circles were assessed and considered as comparative values for spreadability [40].

#### 2.9.4. In Vitro Release Study

The in vitro release study of SIM from different niosomal gel formulations was carried out utilizing the dialysis method [41]. In the dissolution tester, a glass tube open at both ends was mounted and covered with a dialysis membrane (molecular weight cut-off 12,000; Sigma-Aldrich) from one end. The exact weight of 1 g niosomal gel, equivalent to 2 mg SIM, was put on the membrane (donor compartment), which was immersed in a 150-mL beaker containing 30 mL of phosphate-buffered saline pH 6.8 (receptor compartment). The study was performed at 37 °C ± 0.5 °C for 24 h with constant stirring at 100 rpm. At specific time intervals, samples of 2 mL were drawn from the receptor solution and immediately restored with fresh phosphate-buffered saline to retain the sink conditions. All samples were filtered and evaluated for SIM content by HPLC at λ238 (Thermo Fisher Scientific, Waltham, MA, USA). The study was performed in triplicate, and the percentage of SIM released was represented as an average (±SD). The cumulative % release of SIM was plotted against time. To determine the mechanism of SIM release, analysis of the release data was performed using linear regression according to a zero-order and first-order as well as a Higuchi diffusion model [42].

#### 2.9.5. Ex Vivo Permeation Study

The permeation of SIM from the selected niosomal gel formula F7 and free SIM gel were examined via hairless rat skin using a Franz diffusion cell (Rama Glasswares Co. Pvt. Ltd., Delhi, India). Male albino rats ranging from 100 to 150 g were used to perform this study. The abdominal hair of rats was shaved after applying anesthesia (ether). Briefly, 10% ether was applied to animals by means of a simple method using a cotton ball immersed in ether, followed by inhalation for 15 min [43]. Some precautions were implemented during the procedure, such as effective general ventilation and avoiding any ignition source as ether is flammable [44]. Then, the skin samples were isolated from sacrificed rats and cleaned with Dulbecco’s phosphate-buffered saline (pH 7.4) [45]. The study was conducted as follows. The skin was mounted in the diffusion cell so that the stratum corneum was exposed to the donor cell and the dermis was exposed to the receptor medium. Concisely, one gram of SIM niosomal gel was put on the stratum corneum surface (donor compartment). The receptor chamber contained 100 mL phosphate-buffered saline pH 6.8 maintained at 37 °C ± 0.5 °C for up to 24 h. Three-milliliter aliquots were drawn from the receptor medium at prespecified time intervals (namely 1, 2, 3, 4, 6, 8, 12, and 24 h), and restored with an identical amount of fresh medium. Samples were filtered through a 0.45-µm millipore filter, appropriately diluted, and assessed for SIM concentration by means of HPLC at λ238 (Thermo Fisher Scientific, Waltham, MA, USA).

For each formulation, the cumulative amount of SIM permeated per unit area (µg/cm^2^) was plotted against time (h). The linear portion of the graph’s X-intercept was used to determine the lag time. Permeation parameters such as the steady-state flux (Jss) in µg/cm^2^/h, enhancement index (EI), permeation coefficient (Kp) in cm/h, and diffusion coefficient (D) in mm/ min were computed for each formulation to check the enhancement of SIM permeation as compared to the control.

The steady-state flux was calculated using the equation Jss = D dC/dx, where dC/dx is the concentration gradient and D is the diffusion constant [46]. The concentration gradient in diffusion is often called the driving force, although mechanistically it is not a force. The negative sign means that diffusion occurs in the downward direction in the concentration gradient. The permeation coefficient (Kp; the speed of the drug track across the membrane in cm/h) was computed with the equation Kp = Jss/Co, where Jss is the flux and Co is the donor solution concentration [47]. The diffusion coefficient (D) was calculated from the lag time (Tlag) with the following equation [48]:

$$\mathrm{Tlag}=\frac{e^{2}}{6\times D}$$

where e was the thickness, controlled and given by the supplier (thickness 5 mm).

### 2.10. In Vivo Bioavailability Study

An in vivo study was performed to compare the pharmacokinetic parameters of SIM from SIM gel, SIM niosomal gel formulation F7, and SIM oral suspension. Male white albino rats weighing about 300 g were chosen for the in vivo study. All procedures followed the National Institutes of Health (NIH) Guide for the Care and Use of Laboratory Animal [49]. The animal use was approved by the Research Ethics Committee, Department of Pharmacology and Toxicology, Faculty of Pharmacy, Nahda University, Egypt (Approval No. NUB 011019-10/6/2019). The rats fasted for 12 h before administration, with free access to water. The rats were distributed into four groups; each group contained six rats. The first group (Group I) was treated as a control group and received distilled water; Group II was a positive control group and received oral SIM suspension at a dose of 20 mg/kg [50]. Groups III and IV were test groups, exposed to topical SIM gel and SIM niosomal gel F7, respectively. For test groups, SIM gel and niosomal gel F7 were administered topically at a dose equivalent to 20 mg/kg. Then, 0.5 mL of blood samples were withdrawn from the retro-orbital puncture of each rat at 0.5, 1, 1.5, 2, 3, 4, 6, 8, 10, 12, and 24 h after application. The blood samples were assembled in heparinized tubes, followed by centrifugation at 8000 rpm for 10 min to get the plasma. The obtained plasma samples were kept at −20 °C for further assessment.

### 2.11. Preparation of Samples for Analysis

To prepare the samples for analysis, SIM extraction was performed as previously described by Alakhali [51]. Lovastatin, as an internal standard, was dissolved in an acetonitrile and water mixture (80:20 *v*/*v*) to prepare the concentration of 500 ng/mL. Fifty microliters of this lovastatin solution was put in the glass test tube containing 200 µL of a human plasma sample. About 3 mL of an ethyl acetate and hexane mixture (90:10 *v*/*v*) was added to all the test tubes and vortexed for 30 s, followed by centrifugation for 10 min at 4000 rpm. Then, separation and evaporation were done for the organic layer to dryness under a stream of nitrogen at 40 °C. The obtained dry deposits were solubilized with 200 µL of the acetonitrile and water mixture (80:20 *v*/*v*), followed by vortexing for 15 s to obtain clear solutions. For SIM estimation, 20 μL of this solution was injected into the HPLC.

### 2.12. Chromatographic Conditions

For SIM quantitation, a more sensitive Shimadzu HPLC system equipped with a UV visible detector set at 238 nm, a Thermosil^®^ C-18 column (250 × 4.6 × 10 μ, JascoV-530, Artisan Technology Group, Champaign, IL, USA), and a guard column (4.5 mm internal diameter) were used. The mobile phase was composed of acetonitrile and formic acid 3 mM (51:49 *v*/*v*) [51]. The mobile phase was first filtered through a membrane filter, then degassed and pumped at a flow rate of 1 mL/min. All assays were performed under ambient conditions.

### 2.13. Pharmacokinetic Analysis

Pharmacokinetic parameters from plasma data after the oral and transdermal application were assessed using WinNonlin, version 1.5 (Scientific Consulting, Inc., Cary, NC, USA).

## 3. Statistical Analysis

Data analysis was carried out using ANOVA in the Statistical Package For Social Sciences (SPSS; version 19.0) computer software program (SPSS Inc., Chicago, IL, USA), with *p* < 0.05 as a minimal level of significance. The results are represented as average ± SD.

## 4. Results

### 4.1. Factorial Design

Design Expert^®^ (DE) software was configured to operate the factorial design by administering the deduced data. Based on the introductory experiments on the possibility of formulating SIM niosomes, three factors were considered, and each factor had three levels. The signal-to-noise Ratio (SNR) was estimated to ensure the availability of operating the design space navigation [35]. It was noted that all parameters of EE%, vesicle size, zeta potential, and Q12h had a ratio > 4 (Table 1), which is acceptable. Adjusted R-squared (R2) and predicted R-squared (R2) values were identical for all parameters, as shown in (Table 1).

### 4.2. Characterization of SIM Niosome Formulations

#### 4.2.1. Entrapment Efficiency %

**Figure 1:**
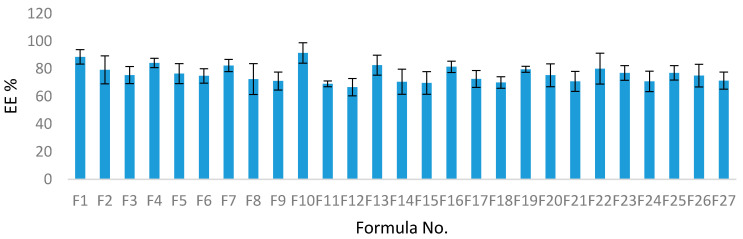
The %EE values of different SIM niosomal formulations.

**Figure 2:**
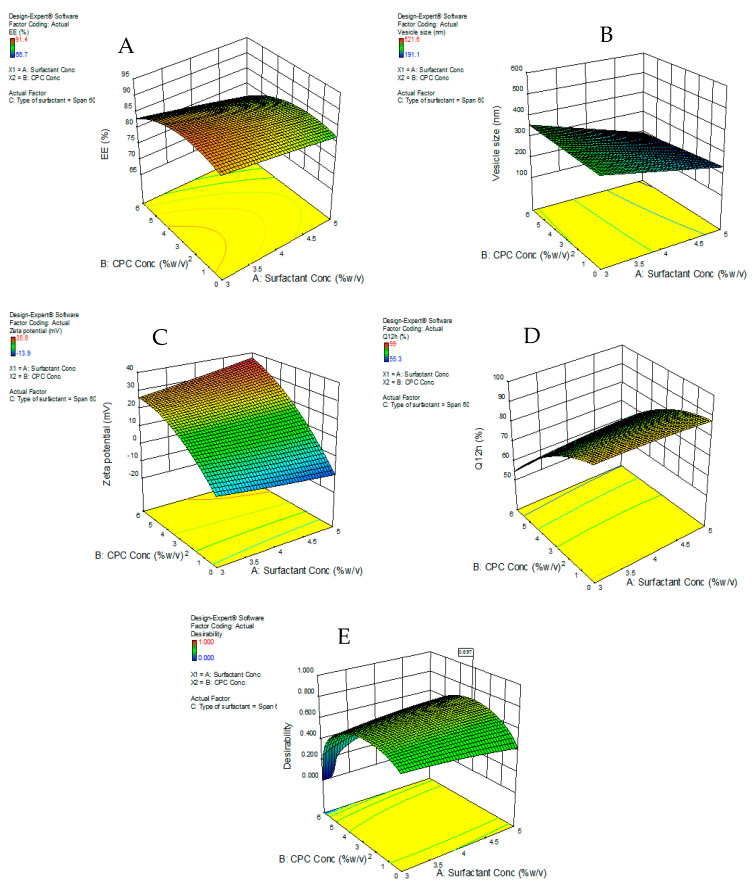
Response surface plot for the effect of CPC concentration (**A**) and type of surfactant (**B**) at the middle levels of the 3rd (surfactant concentration) on (**A**) EE%, (**B**) vesicle size, (**C**) zeta potential, (**D**) Q12h, and (**E**) desirability of the developed niosomal dispersions.

The EE% for the SIM-niosomal formulations ranged between 66.7–91.4% (Table 2) and (Figure 1). Therefore, SIM was effectively encapsulated in the niosomal formulations and this confirms that non-ionic surfactant-based vesicles (niosomes) can be employed as a successful delivery system for lipophilic drugs. The EE% values were considerably influenced by all three independent factors (*p* < 0.05). The effects of the type of surfactant and CPC concentrations on EE% at the middle level of the third independent variable (surfactant concentration) are shown in Figure 2A.

#### 4.2.2. Vesicle Size and Size Distribution

**Figure 3:**
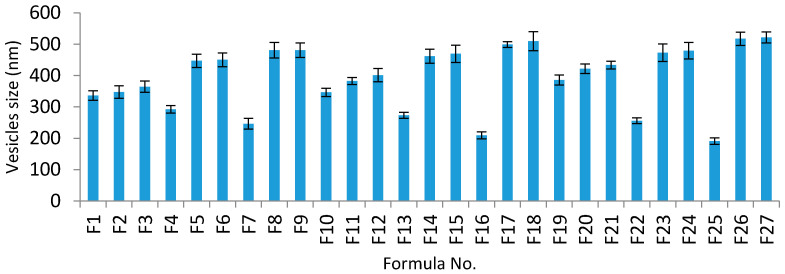
Vesicle size analysis of different SIM niosomal formulations.

Vesicle size is critical for enhancing the transdermal delivery of SIM, and this can be achieved by the formulation of vesicles with an optimized vesicle size. All SIM niosomal formulations showed vesicle sizes ranging from 191.1 to 521.6 nm (Table 2 and Figure 3), confirming a nanosize range.

The vesicle sizes were considerably influenced by all three independent factors (*p* < 0.05). The effects of the type of surfactant and CPC concentrations on vesicle size at the middle level of the third independent variable (surfactant concentration) are shown in Figure 2B.

#### 4.2.3. Zeta Potential

The zeta potential values of all formulations were noted to be in the range of −0.81 to +35.6 mv (Table 2). Increasing CPC concentrations considerably enhanced the zeta potential values. It was also observed that formulations prepared with Tween 80 showed lower zeta potential values than those prepared with Span 60. The three independent variables considerably affected the zeta potential values (*p* < 0.05). The effects of the type of surfactant and CPC concentrations on zeta potential at the middle level of the third independent variable (surfactant concentration) are shown in Figure 2C.

### 4.3. In Vitro Release Study

**Figure 4:**
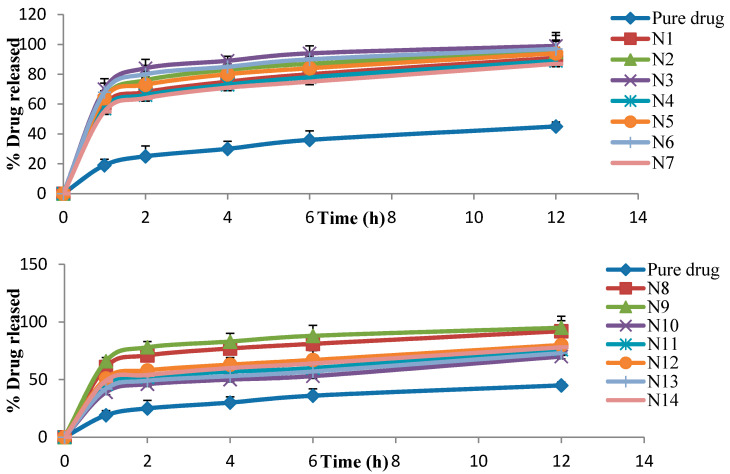

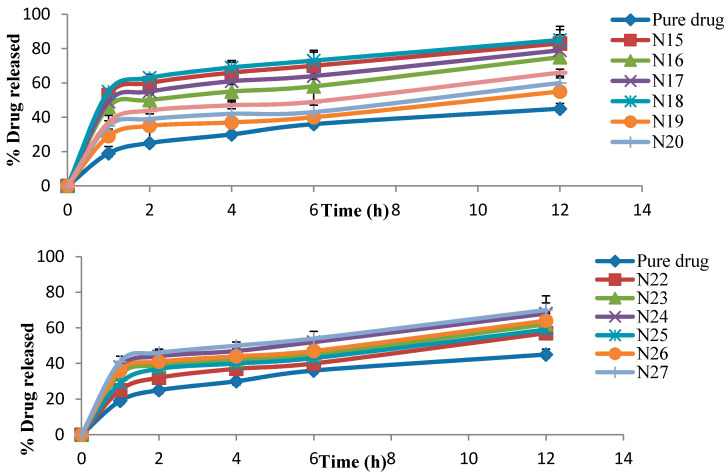
The percentage of drug released from different SIM niosome formulations (N1: N27).

The in vitro release results of SIM from all niosome formulations were examined (Table 2). All formulations revealed a greater release rate of SIM than the pure SIM. The percentage of SIM released after 12 h (Q12h) ranged from 55% to 99%—in contrast, 45% was released from the pure SIM over the same time period (Figure 4).

The in vitro release results were considerably influenced by all three independent factors (*p* < 0.05). The effects of the type of surfactant and CPC concentrations on in vitro release studies at the middle level of the third independent variable (surfactant concentration) are shown in Figure 2D.

It is worth noting that as the CPC concentration increased, Q12h was considerably (*p* < 0.05) decreased. Additionally, all niosome formulations prepared with Cremophor RH 40 gave considerably (*p* < 0.05) higher release rates as compared to those prepared with Tween 80 and Span 60 at the same molar ratios.

### 4.4. Selection of the Optimized SIM Niosomal Formulation

Based on the data analyzed, the responses did not show any significant lack of fit. The desirability approach was used to achieve optimization, which maximizes Y1, Y3, and Y4, and minimizes Y2. The Design Expert^®^ software was selected, as the formulation consisted of a 4% concentration of CPC, the Span 60 type of surfactant, and a 5% concentration of surfactant as the optimum formula (N16), with a desirability of 0.654—the most desirable formula to accomplish this objective (Figure 2E). The responses of the new formula (Table 3) were in near accordance with the predicted values.

**Figure 5:**
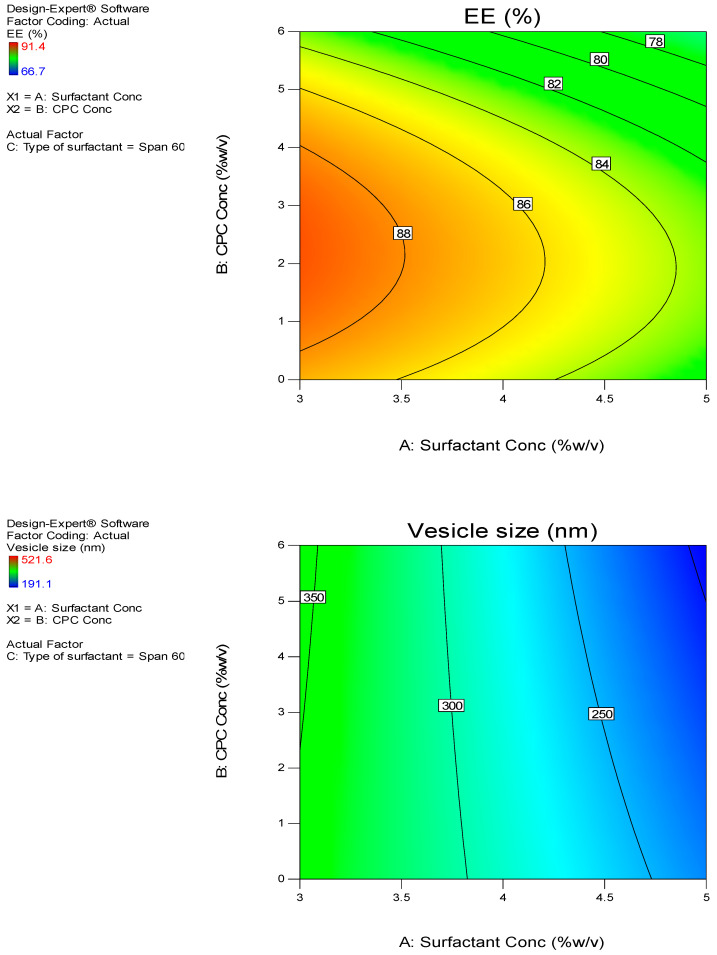

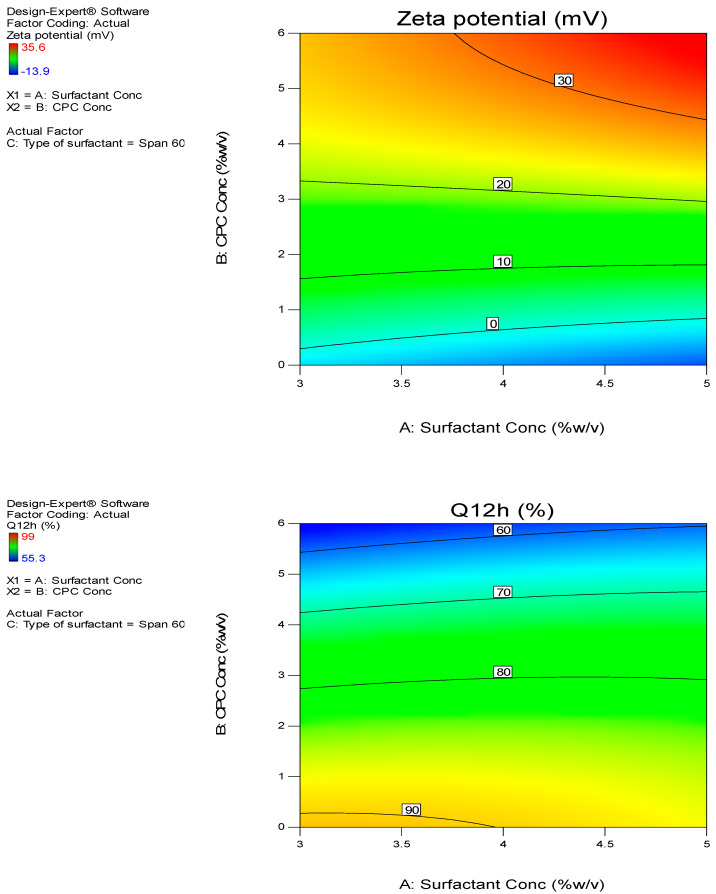
Contour plot of different responses. The figure depicted the contour lines which represented the effect of different formulating factors on different responses.

Additionally, the response surface of all measured responses was appropriately demonstrated by the contour plot (Figure 5). The contour lines showed the effect of different formulating factors on different responses.

### 4.5. Transmission Electron Microscopy

**Figure 6:**
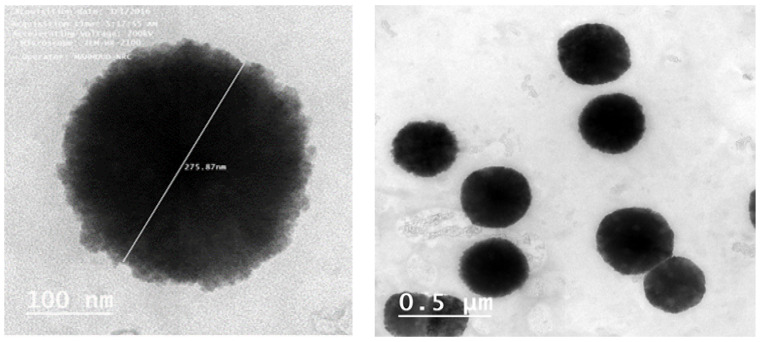
TEM photographs of the optimized SIM niosomal formulation. Images were captured using TEM which showed spherical vesicles with smooth surfaces appearing as black dots, well dispersed and well separate from each other.

The shape and surface characteristics of the optimized formula were observed by means of TEM. The results revealed spherical vesicles with smooth surfaces (Figure 6).

### 4.6. Stability Study

**Figure 7:**
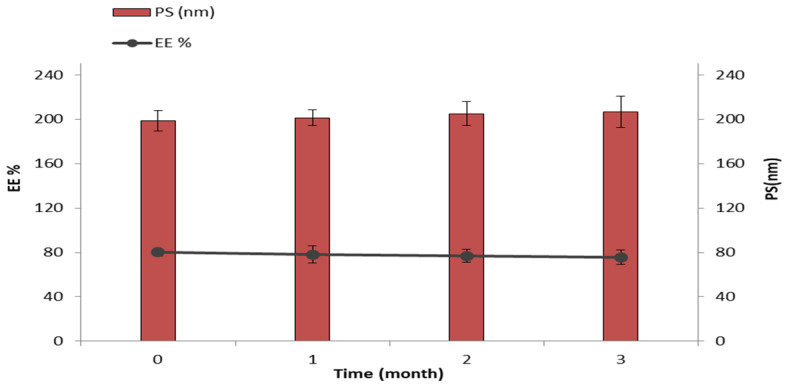
The effect of storage at 4 °C for 3 months on EE% and vesicle size of the optimized SIM niosomal formulation.

The optimum SIM niosomal formulation was stored for 3 months at 40 °C and evaluated for EE% and vesicle size during the storage period. Minor changes were observed in EE% (reduced from 80.21% ± 4.95% to 75.69% ± 5.42%) and vesicle size (increased from 198.62 ± 5.65 nm to 206.66 ± 12.05 nm) over the period of study (Figure 7). These changes proved to be insignificant (*p* > 0.05) in a one-way ANOVA test.

### 4.7. Characterization of SIM-Loaded Niosomal Gel

#### 4.7.1. Physical Parameters

All niosomal gel formulations showed acceptable pharmaceutical properties. The color was transparent to slightly white, and the appearance was smooth without any phase separation. All formulations were homogeneous and clear without any particulate matter. These properties are consistent with the ideal requirements of a topical gel [52].

#### 4.7.2. pH Measurements

The pH of all SIM niosomal gel formulations ranged between 6.1–6.9 (Table 4), which is considered an acceptable range for topical preparations as it is within the physiological skin pH [53]. It would therefore not cause any irritation upon administration to the skin surface.

#### 4.7.3. Spreadability

Spreadability plays an essential role in patient satisfaction and the easy administration of gel to the skin. For a gel to be acceptable, it should spread rapidly. All gel formulations showed better spreadability than the control gel. Spreadability values ranged from 2.3 cm to 4.7 cm (Table 4), indicating gel spreadability with a small amount of shear [40]. F7 showed the best spreadability.

#### 4.7.4. In Vitro Release Study

**Figure 8:**
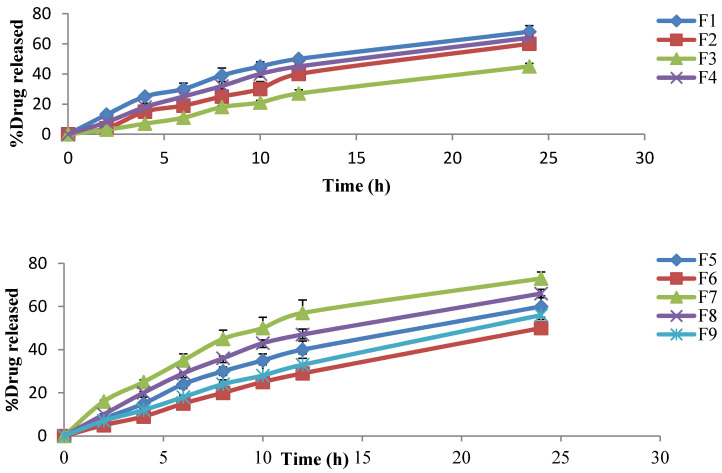
The percentage of drug released from different niosomal gel formulations.

The cumulative percentage of SIM released from different niosomal gel formulations was found to range from 3.6–16.4% after the first 2 h, increasing to 27.3–57.6% after 12 h, and reaching 45.6–73.5% after 24 h, compared with the release of 68.7% from the optimized SIM niosomal dispersion after 12 h. It was also noted that Na CMC gels showed the highest drug release compared to HPMC H15 and carpobol 940 gels (Table 4 and Figure 8).

The mathematical analysis of the SIM release data showed that the release of SIM from different gel formulations followed the Higuchi diffusion model (Table 5) [54].

F7 showed the highest release rate, as 16.4% of SIM was released after 2 h, 57.6% was released after 12 h, and 73.5% was released after 24 h. F7 also showed acceptable physical properties and high spreadability. Therefore, F7 was chosen for the ex vivo permeation study.

#### 4.7.5. Ex Vivo Permeation Study

An ex vivo permeation study was conducted to explore the in vivo performance of SIM-loaded niosomal gel (F7) in comparison to SIM gel.

**Figure 9:**
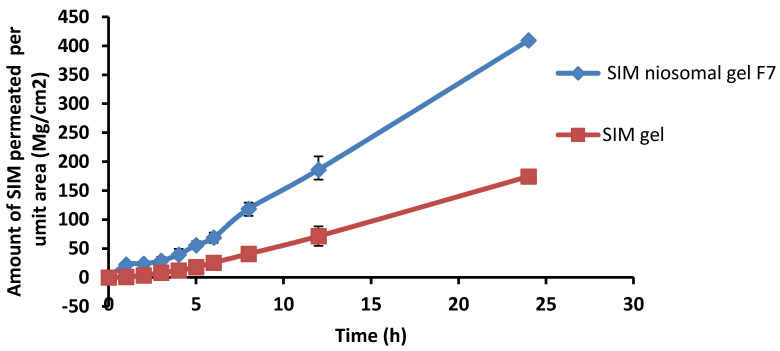
Skin permeation data for SIM niosomal gel formula F7 compared to SIM gel over 24 h.

The amount of SIM permeated from the loaded niosomal gel was found to be 23.6 µg/cm^2^ after the first 2 h, increasing to 186.2 µg/cm^2^ after 12 h, and reaching 409.5 µg/cm^2^ after 24 h. Although the amount permeated from SIM gel was 3.6 µg/cm^2^ after the first 2 h, this increased to 71.3 µg/cm^2^ after 12 h, and reached 174.4 µg/cm^2^ after 24 h (Figure 9). Thus, the SIM-loaded niosomal gel permeated to a greater extent than the SIM gel, which emphasizes the efficacy of niosomes as a promising system for the transdermal delivery of drugs.

The permeation parameters for SIM-loaded niosomal gel and SIM gel across rat skin was calculated (Table 6). It was clear that the transdermal flux of SIM niosomal gel was 13.77 ± 1.79 µg/cm^2^ h, compared to 5.68 ± 1.4 µg/cm^2^ h for the SIM gel. All permeation parameters confirmed the superiority of the niosomal gel formulation compared to the SIM gel. The enhancement ratio of SIM-loaded niosomal gel was 2.42-fold greater than SIM gel.

#### 4.7.6. In Vivo Bioavailability Study

**Figure 10:**
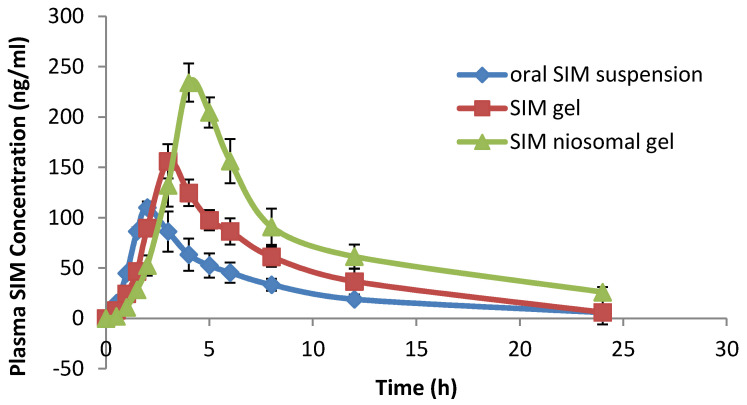
Plasma drug concentrations–time profiles of SIM following administration of niosomal gel formula, SIM gel, and oral SIM suspension.

Based on the previous evaluations, niosomal gel formulation F7, containing 2% Na CMC, was nominated for the in vivo study. The plasma drug concentration at different time intervals after administration of SIM-loaded niosomal gel F7, SIM gel, and oral SIM suspension was determined and is represented in Figure 10.

Various pharmacokinetic parameters were calculated, such as maximum plasma concentration (Cmax), peak time (Tmax), area under the curve (AUC), and mean residence time (MRT), as provided in Table 7.

It is clear from the data that the SIM-loaded niosomal gel showed significantly greater Cmax and Tmax values (*p* ˂ 0.05) as compared to SIM gel and the SIM suspension, indicating enhanced delivery of SIM from niosomes in a controlled manner.

Additionally, the values of AUC_0–∞_ and MRT_0–∞_ of SIM-loaded niosomal gel were 2-fold and 1.8-fold greater than the oral SIM suspension, respectively, and 1.5-fold and 1.49-fold higher than the SIM gel. The differences were significant (*p* < 0.05), as shown in Table 8.

## 5. Discussion

Nanovesicular systems are attracting wide attention for the transdermal delivery of many drugs. Examples of these nanovesicular systems are liposomes, niosomes, transferosomes, and ethosomes. Liposomes—lipid vesicles with a combination of phospholipids and cholesterol—have the advantages of being biodegradable and nontoxic, and are used to encapsulate both hydrophilic and lipophilic drugs. However, their ability to permeate the skin is very low due to their rigidity [55]. In this regard, other nanovesicular systems (modified flexible liposomes) have been prepared to enhance the permeation through the skin, including niosomes (non-ionic surfactant vesicles), transferosomes (ultraflexable liposomes containing phospholipids and edge activators), and ethosomes (flexable liposomes containing phospholipids and ethanol) [55,56]. However, niosomes are more advantageous than ethosomes as they exhibit a better release and are more stable and safer. Moreover, niosomes deliver the drugs more efficiently into the deep layers of the skin in contrast to ethosomes, which show more deposition of the drugs within the skin with low permeation [57].

In our study, SIM-loaded niosomes were prepared and characterized for different parameters such as EE%, vesicle size, zeta potential, and percentage of drug released.

The EE% values are represented in Table 2. It was clear from the data that increasing the concentration of CPC from 0% (N1 88.4%) to 4% (N10-91.4%) increased the EE%. This may be due to the highly hydrophilic nature of the bilayer membrane, so the higher water intake of the vesicles’ bilayers may result in higher entrapment of the drug. This finding is in agreement with the results formerly reported by Hashim et al. [58]. It was also found that all niosomes prepared with Span 60 showed higher EE% values than those prepared with Tween 80 and Cremophor RH 40, which can be possibly attributed to many factors [59]. (a) The hydration temperature employed in niosome preparation must be higher than the transition temperature of the system and this results in niosomes that show less permeability and high EE%. Span 60 shows the highest phase transition temperature (higher than 50 °C) [59] in comparison to Tween 80 and Cremophor RH40, so Span 60 had a positive impact on EE%. (b) The alkyl chain length of surfactant exerts a noticeable effect on the niosomal membrane permeability such that as the alkyl chain length increases, EE% also increases due to the reduction in vesicle permeability. Therefore, the surfactant that has a longer alkyl chain will result in higher entrapment. Span 60 has an alkyl chain longer than those of Tween 80 and Cremophor RH40, thus it produced niosomes with higher EE% [60]. (c) The alkyl chain length affects the surfactant’s Hydrophilic lipophilic balance (HLB) value, which sequentially clearly affects the EE%. The longer the alkyl chain, the lower the HLB of the surfactant, and the higher will be the EE%. Span 60 has the lowest HLB (4.7) [61] as compared to Tween 80 and Cremophor RH40 (HLB > 12) [62].

The formula N10 prepared from Span 60 showed the highest EE%, at 91.4% (Figure 1). Thus, the EE% values of SIM niosomes formulations could be arranged as Span 60 > Tween 80 > Cremophor RH 40.

The vesicle size values are presented in Table 2. It was found that enhancing the concentration of CPC considerably increased the average vesicle size, and this may be attributed to the higher amount of SIM encapsulated in the niosomal vesicles. Additionally, the inclusion of CPC (a positive charge inducer) causes an increase in the hydrophilicity of the vesicles’ membranes; thus, greater water uptake by the vesicle membranes may cause an increase in the vesicle size. These results are in agreement with the research by Junyaprasert et al. [63]. It was also noted that the type of surfactant had a noticeable impact on vesicle size, and the niosomes prepared with Span 60 had smaller vesicle sizes than those prepared with Tween 80 and Cremophore RH40, respectively. This may have resulted from the reduction in the surface free energy associated with the increased lipophilicity (low HLB) of the surfactant [64]. Furthermore, niosomes’ vesicle sizes decreased on increasing the Span 60 concentration because of the increased lipophilicity of the surfactant. This causes a reduction in the surface free energy and vesicle size [65]. These results are in accordance with those of Hashim et al. [58]. Thus, the vesicle size of SIM niosomes formulations could be arranged as Cremophor RH 40 > Tween 80 > Span 60.

The PDI, an indicator of the vesicle size distribution, [66] was found to be in the range of 0.06 (N24)–0.30 (N25), indicating low variation in the vesicle sizes.

Zeta potential affects system stability, as it measures the extent of electrostatic repulsion and attraction between particles. If the zeta potential values are too high, repulsion will occur, and the stability of the system will be high. Vesicles are stable if the zeta potential values above +30 or below −30 mv [67] Inducing positive charges (CPC) at the surface of vesicles not only affects niosomal physicochemical features and stability, but also enhances transdermal permeation via its interaction with biological membranes [20,68].

It was noted from the data that increasing CPC concentrations considerably enhanced the zeta potential values. A possible interpretation for this enhancement is that CPC produces positive charges on the vesicles’ surface, causing strong electrostatic repulsions between the vesicles [20]. It was also observed that formulations prepared with Tween 80 showed lower zeta potential values than those prepared with Span 60, and this is because of the larger hydrophilic head group of Tween 80 compared to Span 60. This result is in accordance with the previous report by Waddad et al. [69]. Therefore, formulations containing Span 60 were more stable. Thus, the zeta potential values of SIM niosome formulations could be arranged as Span 60 > Tween 80 > Cremophor RH 40.

These findings confirm the high stability of the niosomes, and these positively charged niosomes result in strongly enhanced SIM permeation during transdermal delivery [68].

The in vitro release profiles showed a greater release rate of SIM from all formulations than the pure SIM and this may be due to the solubilizing effect of niosomes, which cause improvements in the drug release [70]. Additionally, as the CPC concentration increased, Q12h was considerably (*p* < 0.05) decreased, and this may be attributed to the high repulsion forces and high stability of charged niosomes [58]. In addition, increasing the CPC concentration resulted in bigger vesicles with a small surface area subjected for dissolution medium.

All niosome formulations prepared with Cremophor RH 40 gave considerably (*p* < 0.05) higher release rates as compared to those prepared with Tween 80 and Span 60 at the same molar ratios. This could be clarified on the basis that niosomes show a release pattern dependent on the alkyl chain length, and the greater the alkyl chain length, the lesser the rate of drug release [71]. Span 60 has the longest saturated alkyl chain as compared to Tween 80 and Cremophore RH40, thus it exhibited the slowest drug release. This result is in accordance with Mazyed et al. [72]. Additionally, the surfactant transition temperature affects the drug release rate from the niosomes. It was noted that enhancing the transition temperature reduces the rate of drug release from vesicles [73]. Span 60 has the highest phase transition temperature (50 °C) in comparison to Tween 80 and Cremophor RH40, so it showed the slowest drug release. Thus, the release pattern of SIM could be arranged as Cremophor RH 40 > Tween 80 > Span 60.

The TEM photographs of the optimized formula revealed the absence of any aggregations, indicating that the optimized formulation was well dispersed and physically stable due to the small and homogenous vesicle size.

The optimum SIM niosomal formulation showed good stability data, and this may be due to the vesicles’ rigidity and/or the capability of the vesicles to retain the drug [74].

The optimized SIM niosomal formulation was integrated into different gel bases (Carbapol 940, HPMC H15, and Na CMC), then these were evaluated for their in vitro release. It was noted that the niosomal gel formulations showed a slow release as compared to the optimized niosomal dispersion and this may be due to the release from niosomal vesicles, along with the diffusion of drug via the polymer network channel structures of the gel. Thus, the encapsulated SIM could permeate slowly from the niosomes’ vesicles into the network structure of the gel. This result is in accordance with the work of Barakat et al. [75], indicating a controlled release pattern for niosomal gel formulations. Additionally, Na CMC gels showed the highest drug release compared to HPMC H15 and Carpobol 940 gels. This could be explained on the basis of the high swelling action and increased the solubility of Na CMC in the dissolution medium, thus resulting in an enhancement in the transfer of the drug outside the matrix. However, Carbopol gel formulations showed the slowest release, and this may be due to attraction between the positively charged niosomal nanovesicles and the negative nucleus of Carbopol 940. The release pattern of SIM from gel formulations could be ranked as Na CMC > HPMC H15 > Carpobol 940.

SIM-loaded niosomal gel showed higher permeation parameters than the SIM gel. The capability of niosomes to augment drug permeation throughout the skin may be attributed to many mechanisms [76]. Firstly, it may be attributed to the fusion of niosomes with the stratum corneum intercellular lipid, causing it to become looser and more permeable. Secondly, the vesicles improve the penetration by decreasing the stratum corneum barrier characteristics. Thirdly, the non-ionic surfactant has a permeation-enhancing effect [77].

Regarding the pharmacokinetic parameters, SIM-loaded niosomal gel showed higher Cmax values (*p* ˂ 0.05) as compared to the SIM gel and SIM suspension, and this could be attributed to the existence of non-ionic surfactants (a main component in the structure of niosomes), which have a penetration-enhancing effect [77]. In addition, the T_max_ of SIM-loaded niosomal gel was higher than that of the SIM gel and SIM suspension (*p* ˂ 0.05), and this could be due to the controlled release of SIM from the vesicles’ lipid bilayers, which act as a rate-controlling barrier for the release of drugs [78]. However, the value of T_max_ was still lower than the expected value. This unexpected result of a low T_max_ value compared to the slow in-vitro release of SIM niosomal gel may be due to interaction between the niosomal membrane and serum proteins (opsonins) that cause lysis of niosomes vesicles and consequently release the encapsulated SIM into the blood circulation [79]. In addition, low density lipoproteins (LDL) and high-density lipoproteins (HDL) have negative effects on the stability of niosomal membrane lipid contents, causing rapid release of the drug from the niosome vesicles [80]. These results are in agreement with previously reported results [81].

Finally, the values of AUC_0–∞_ and MRT_0–∞_ of the SIM-loaded niosomal gel were greater than the oral SIM suspension and SIM gel, indicating that the niosomal gel delivered the SIM to the systemic circulation more efficiently than the SIM gel and oral SIM suspension. The improvement of SIM delivery from SIM-loaded niosomal gel was mainly due to the avoidance of the problems related to drug absorption from the gastrointestinal tract, especially the first pass metabolism.

## 6. Conclusions

A new SIM niosomal delivery system was prepared and evaluated in this work in order to overcome the poor bioavailability of SIM. Many factors, such as surfactant content, surfactant type, and charge inducing agent, were optimized to obtain an optimum preparation with high EE%. The prepared niosomal formulations were evaluated for different parameters such as EE%, zeta potential, vesicle size, and in vitro drug release. The surface features of the optimized formulation were evaluated by means of TEM and ultimately incorporated into different hydrogel bases and evaluated for different characteristics. Then, the best niosomal gel formulation was chosen for pharmacokinetic study in comparison with SIM gel and oral SIM suspension. The in vivo study suggested that niosomes can prolong the drug release, with higher bioavailability in comparison with SIM gel and SIM suspension. Ultimately, we can conclude that SIM-loaded niosomal gel is a potential new nanocarrier for transdermal SIM delivery into the systemic circulation. Our plan for the future is to compare the transdermal delivery of SIM-loaded niosomes with other deformable nanovesicles such as transferosomes and ethosomes.

## Figures and Tables

**Table 1:** Composition of different coded values in 3^3^ full factorial design for optimization of simvastatin (SIM) niosomes.

Independent Variable		Code Value					
		−1		0		1	
surfactant concentration *w*/*v* % (X1)		3		4		5	
Type of surfactant (X2)		Span 60		Tween 80		Cremophor RH 40	
CPC concentration (X3)		0		4		6	
Dependent variables	R2	Adjusted R2	Predicted R2	Constraints	*p*-value	F value	Adequate precision
Y1: EE%	0.6933	0.6677	0.6118	Maximize	0.0001	27.13	10.014
Y2: Vesicles size (nm)	0.9954	0.9899	0.9765	Minimize	0.0001	183.71	43.525
Y3: Zeta potential (mV)	0.7795	0.7611	0.7209	Maximize	0.0001	312.88	11.878
Y4: Q12h (%)	0.9992	0.9983	0.996	Maximize	0.0001	1082.13	103.883

CPC, cetyl pyridinium chloride; EE%, percent of entrapment efficiency; Q12h, cumulative release after 12 h.

**Table 2:** 3^3^ full-factorial design of SIM-loaded niosomes with responses (vesicle size, EE%, % drug released, and zeta potential).

Formulation Code	X1	X2	X3	Vesicles Size ^a^ (nm)	% Entrapment	% Drug Released ^a^	Zeta Potential ^a^	PDI ^a^
					Efficiency ^a^			
N1	−1	−1	−1	336.1 ± 15.20	88.6 ± 5.25	91.1 ± 2.2	−1.39 ± 0.15	0.12
N2	−1	0	−1	347.5 ± 20.15	69.1 ± 10.20	94.2 ± 1.26	−0.81 ± 0.11	0.14
N3	−1	1	−1	364.6 ± 18.26	66.7 ± 6.15	99.0 ± 0.94	−4 ± 0.02	0.09
N4	0	−1	−1	292.3 ± 12.25	82.6 ± 3.45	90.3 ± 3.11	−6.7 ± 0.06	0.13
N5	0	0	−1	447.3 ± 21.15	70.6 ± 7.25	94.4 ± 1.11	−1.07 ± 0.02	0.11
N6	0	1	−1	450.2 ± 22.15	69.7 ± 5.20	97.5 ± 4.38	−6.2 ± 0.03	0.21
N7	1	−1	−1	246.4 ± 16.9	81.4 ± 4.41	87.1 ± 3.04	−13.9 ± 0.00	0.15
N8	1	0	−1	480.9 ± 24.73	72.6 ± 11.20	92.1 ± 1.04	−1.89 ± 0.02	0.16
N9	1	1	−1	481.2 ± 23.15	70.1 ± 6.56	95.4 ± 3.01	−9.3 ± 0.01	0.1
N10	−1	−1	0	346.4 ± 13.1	91.4 ± 7.41	70.6 ± 6.02	+22.5 ± 2.1	0.12
N11	−1	0	0	382.8 ± 11.25	79.2 ± 2.15	76.2 ± 2.01	+12.7 ± 3.10	0.16
N12	−1	1	0	401.3 ± 21.20	75.4 ± 6.3	80.3 ± 4.01	+9.3 ± 1.5	0.14
N13	0	−1	0	273.2 ± 9.30	84.2 ± 7.2	73.6 ± 2.01	+27.7 ± 3.60	0.12
N14	0	0	0	461.8 ± 22.1	76.5 ± 9.15	78.7 ± 1.01	+15 ± 2.41	0.08
N15	0	1	0	469.3 ± 27.20	74.8 ± 8.15	83.1 ± 2.02	+12.8 ± 3.01	0.11
N16	1	−1	0	209.53 ± 11.2	82.3 ± 4.15	75.5 ± 4.03	+32.9 ± 2.13	0.15
N17	1	0	0	499.01 ± 9.1	72.5 ± 6.1	79.4 ± 2.00	+19 ± 4.30	0.19
N18	1	1	0	509.5 ± 30.3	71.1 ± 4.20	85.7 ± 1.7	+17.5 ± 1.04	0.17
N19	−1	−1	1	385.7 ± 16.25	79.6 ± 2.20	55.3 ± 1.09	+25 ± 2.53	0.16
N20	−1	0	1	421.6 ± 15.35	75.3 ± 8.30	60.1 ± 2.30	+14 ± 1.02	0.18
N21	−1	1	1	433.5 ± 12.26	70.8 ± 7.25	66.0 ± 4.25	+10.9 ± 2.02	0.10
N22	0	−1	1	255.9 ± 9.2	80.1 ± 11.2	57.5 ± 3.01	+28.3 ± 3.01	0.13
N23	0	0	1	472.9 ± 28.20	76.9 ± 5.3	63.4 ± 4.11	+17.6 ± 4.01	0.15
N24	0	1	1	479.3 ± 26.15	70.9 ± 7.40	68.2 ± 2.07	+17.2 ± 2.11	0.06
N25	1	−1	1	191.1 ± 10.20	77.03 ± 5.22	59.1 ± 1.04	+35.6 ± 1.01	0.30
N26	1	0	1	517.3 ± 21.3	75.1 ± 8.25	64.6 ± 3.33	+22.8 ± 3.41	0.15
N27	1	1	1	521.6 ± 17.25	71.4 ± 6.25	69.3 ± 1.12	+24.6 ± 4.01	0.13

^a^ Values represented as mean ± SD (*n* = 3). PDI, polydispersability index.

**Table 3:** Predicted and experimental values of the optimized SIM niosomal formulation (*n* = 3).

Solution	CPC Concentration	Type of Surfactant	Conc of Surfactant	Y1	Y2	Y3	Y4	Desirability
Predicted	4	Span 60	5	83.03	211.87	18.82	74.67	0.654
Experimental	4	Span 60	5	80.21	198.62	36.76	68.78	0.654
Bais %	-	-	-	0.03	0.05	0.12	0.08	-

%Bias = (predicted value − experimental value)/experimental value.

**Table 4:** SIM-loaded niosomal gel formulations with responses (clarity, homogeneity, pH, spreadabilty, and % drug released).

Formulation Code	Polymer Conc (*w*/*w*)	Polymer Type	Clarity	Homogeneity	pH Values ^a^	Spreadabilty ^a^	% Drug Released ^a^ after 24 h
F1	2	HPMC	+++	Homogenous	6.8 ± 0.01	2.7 ± 0.17	68.3 ± 0.351
F2	3	HPMC	+++	Homogenous	6.5 ± 0.03	2.3 ± 0.34	60.1 ± 0.305
F3	4	HPMC	++	Homogenous	6.3 ± 0.02	2.4 ± 0.35	45.6 ± 0.032
F4	2	Carbopol 940	+++	Homogenous	6.9 ± 0.11	3.7 ± 0.20	64.2 ± 0.172
F5	3	Carbopol 940	+++	Homogenous	6.4 ± 0.12	3.3 ± 0.15	60.6 ± 0.136
F6	4	Carbopol 940	+++	Homogenous	6.2 ± 0.10	3.1 ± 0.10	50.3 ± 0.072
F7	2	Na CMC	+++	Homogenous	6.6 ± 0.02	4.7 ± 0.17	73.5 ± 0.050
F8	3	Na CMC	+++	Homogenous	6.4 ± 0.01	3.2 ± 0.24	66.7 ± 0.044
F9	4	Na CMC	++	Homogenous	6.1 ± 0.00	4.1 ± 0.68	56.3 ± 0.014

^a^ Values represented as mean ± SD (*n* = 3); clear: ++, very clear (glassy): +++.

**Table 5:** Kinetic analysis of SIM-loaded niosomal gel.

Figure	Correlation Coefficient (R2)		
	Zero	First	Higuchi
F1	0.854	0.901	0.967
F2	0.792	0.812	0.981
F3	0.866	0.751	0.968
F4	0.819	0.869	0.959
F5	0.793	0.877	0.971
F6	0.855	0.761	0.962
F7	0.901	0.695	0.957
F8	0.793	0.828	0.945
F9	0.892	0.741	0.961

**Table 6:** In vitro permeation parameters of SIM-loaded niosomal gel formulations versus SIM gel.

Figure 1	Lag Time (min)	Jss (µg/cm^2^ h)	Kp (cm/h)	D (mm/min)	EI
SIM niosomal gel F7	23.14 ± 4.92	13.77 ± 1.79	0.0138 ± 0.0018	0.857	2.42
SIM gel	82.42 ± 11.31	5.68 ± 1.40	0.0057 ± 0.0014	0.050	-

Jss: steady state flux, Kp: permeation coefficient, D: Diffusion coefficient, EI: enhancement index.

**Table 7:** Pharmacokinetic parameters of SIM in plasma after administration of SIM-loaded niosomal gel F7, SIM gel, and SIM suspension.

Pharmacokinetic Parameters	Formula		
	OralSIM Suspension	SIM Gel	SIM Niosomal Gel (F7)
C_max_	110.01 ± 10.2	150.6 ± 11.64	230.80 ± 17.27
T_max_	2.06 ± 0.13	3.33 ± 0.24	4.5 ± 0.62
T_1/2_	4.5 ± 0.37	7.2 ± 0.85	10.65± 1.20
AUC_0–∞_	990.94 ± 34.29	1300.04 ± 85.73	1930.06 ± 117.19
MRT_0–∞_	9.17 ± 1.35	11.1 ± 1.65	16.62 ± 2.06

Data represented as mean  ±  SD (*n*  =  6).

**Table 8:** Statistical analysis of pharmacokinetic parameters of SIM in plasma after administration of SIM-loaded niosomal gel, SIM gel, and SIM suspension.

Pharmacokinetic Parameters	Df	MS	F Value	*p*-Value	Significance
C_max_ (ng/mL)	2	209.91	118.683	1.2417 × 10^−8^	Sig.
T_max_ (h)	2	4.334	5.836	0.016962	Sig.
T_1/2_	2	9.462	12.436	0.004265	Sig.
AUC_0–∞_ (ng h/mL)	2	11,290.30	328.288	3.34 × 10^−11^	Sig.
MRT_0–∞_ (h)	2	71.418	20.9834	0.00012	Sig.

## Data Availability

Not applicable.
